# Expiratory Flow Limitation Definition, Mechanisms, Methods, and Significance

**DOI:** 10.1155/2013/749860

**Published:** 2013-03-28

**Authors:** Claudio Tantucci

**Affiliations:** Department of Experimental and Clinical Sciences, University of Brescia, 1a Medicina, Spedali Civili, 25123 Brescia, Italy

## Abstract

When expiratory flow is maximal during tidal breathing and cannot be increased unless operative lung volumes move towards total lung capacity, tidal expiratory flow limitation (EFL) is said to occur. EFL represents a severe mechanical constraint caused by different mechanisms and observed in different conditions, but it is more relevant in terms of prevalence and negative consequences in obstructive lung diseases and particularly in chronic obstructive pulmonary disease (COPD). Although in COPD patients EFL more commonly develops during exercise, in more advanced disorder it can be present at rest, before in supine position, and then in seated-sitting position. In any circumstances EFL predisposes to pulmonary dynamic hyperinflation and its unfavorable effects such as increased elastic work of breathing, inspiratory muscles dysfunction, and progressive neuroventilatory dissociation, leading to reduced exercise tolerance, marked breathlessness during effort, and severe chronic dyspnea.

## 1. Definition

Expiratory (air) flow limitation (EFL) during tidal breathing is a well-defined, mechanical pathophysiological condition occurring, either during physical exercise or at rest, before in supine and later on in sitting-standing position, when expiratory flow cannot be further increased by increasing expiratory muscles effort (i.e., by increasing pleural and alveolar pressure) because it is maximum at that tidal volume [1]. In other words, under the prevailing conditions, the respiratory system is globally limited as flow generator even during tidal expiration, and greater expiratory flow rates may be achieved just by increasing operating lung volumes, (i.e., moving progressively the end-expiratory lung volume (EELV) towards total lung capacity). In fact, the volume-related decrease of airway resistance and increase of elastic recoil are the only effective mechanisms to obtain higher expiratory flows in case of EFL [2].

**Figure 1:**
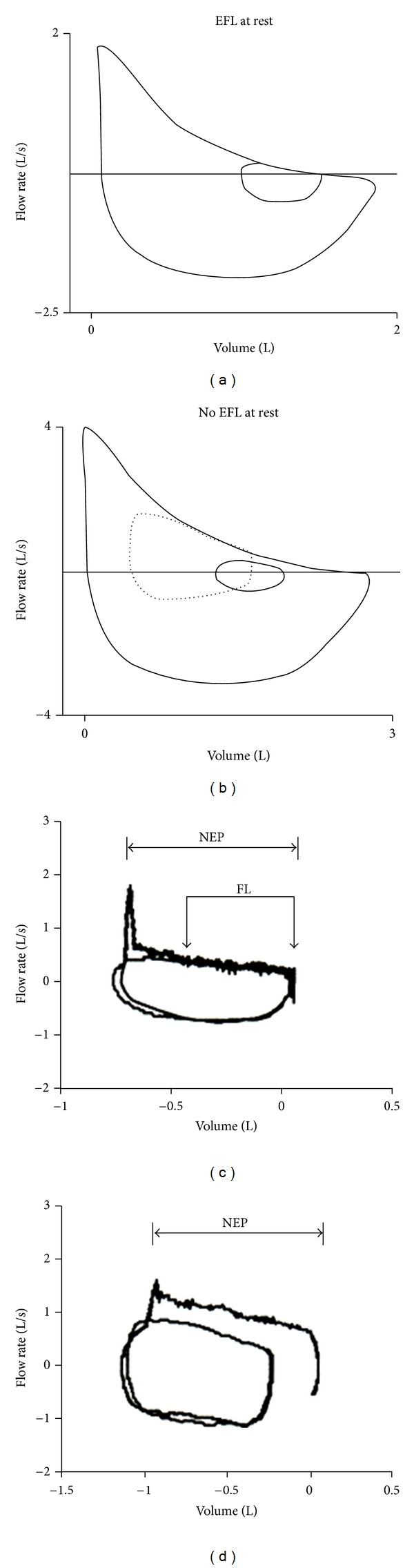
Maximal and tidal flow-volume curve in two representative COPD patients: one with airflow reduction and tidal expiratory flow limitation (EFL) at rest (a), the other only with airflow reduction at rest and potential EFL during exercise (b). The NEP application at rest does not increase expiratory flow in the first patient (c), while eliciting greater expiratory flow in the second one (d).

As a consequence, the term airflow limitation widely used to indicate the abnormal decrease of maximal expiratory flow rates at a given lung volume, as compared to predicted (i.e., airflow reduction or airflow obstruction), is inappropriate and should not be adopted unless the condition previously described is present (Figure 1).

## 2. Mechanisms of EFL

Several mechanisms may contribute to the EFL development by reducing the expiratory flow reserve in the tidal volume range.

The age-related increment of closing volume and closing capacity may induce in the elderly the closure of dependent small airways above EELV, causing a functional amputation of lung volume with consequent decrease in maximal expiratory flow rates corresponding to tidal volume [3]. Actually, the lung senescence may predispose to EFL, especially in the supine position and in small sized, overweight women.

When supine, the relaxation volume of the respiratory system (*V*_*r*_) is lower as a result of gravitational forces, and usually EELV decreases with recumbency [4]. Since the maximal flow-volume curve denotes minimal variation by assuming the supine position [5], this body position predisposes to EFL because tidal breathing occurs at lower lung volumes where maximal expiratory flow rates are necessarily less.

Breathing at low-lung volume (near residual volume), as frequently observed in great and massive obesity, chronic congestive heart failure, and sometimes in restrictive lung and chest wall disorders, intrinsically reduces the maximal expiratory flow rates in the tidal volume range, facilitating the EFL occurrence, mainly in the supine position.

Higher ventilatory requirements with larger tidal volume (for similar respiratory rate and expiratory time), faster respiratory rate and shorter expiratory time (for similar tidal volume), or both, as expected during exercise or observed even at rest in various conditions, do increase mean tidal expiratory flow and reduce expiratory flow reserve during tidal breathing, making easier to have EFL.

**Figure 2:**
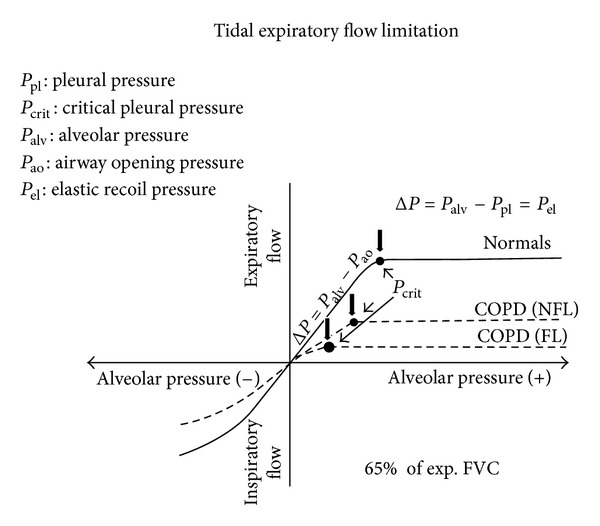
Isovolume (low-lung volume) flow-pressure relationship in normal subjects, COPD without expiratory flow limitation (NFL) and COPD with expiratory flow limitation (FL). In any case, after *P*_crit_, expiratory flow does not increase further on, and its driving pressure becomes *P*_el_. In COPD patients with high airflow resistance and very low *P*_el_, the *P*_crit_ occurs early, limiting expiratory flow in the tidal volume range.

On the other hand, EFL is linked inescapably to the presence of airflow reduction, no matter what is the prevailing mechanism (increased airway resistance, augmented cholinergic bronchial tone, decreased lung elastance, airway-parenchyma uncoupling, and airways collapsibility) in the obstructive lung diseases such COPD (Figure 2), chronic asthma, cystic fibrosis, constrictive bronchiolitis [6, 7]. In this respect, predominant reduction of maximal expiratory flow rates at lower lung volumes appears more crucial in promoting EFL. However, the site where the system becomes entirely flow-limited and flow limiting segment develops can be located centrally or peripherally. When EFL originates in the peripheral airways, it is mainly due to the viscous, density-independent, flow-limiting mechanism, while the speed wave, density-dependent, flow-limiting mechanism is substantially involved, when the EFL originates in the central airways [8]. 

Therefore, aging, body position, exercise, hyperpnea-tachypnea, low-volume breathing, or airflow reduction represents, alone or more often combined together, the main factors that favor the development of EFL in humans.

## 3. Methods for EFL Detection

Classically, flow limitation may be detected looking at isovolume pleural (or alveolar) pressure-flow relationship, and it occurs, when expiratory flow rate does not change (or even is reduced) despite the increasing pleural (alveolar) pressure [9]. Therefore, if increasing pleural pressure at lung volume corresponding to tidal breathing induces no change in expiratory flow, EFL is documented. Comparison between full (or partial) maximal and resting flow-volume loops has been used to detect EFL which is assumed when expiratory tidal flow impinges on or is even greater than maximal expiratory flow at the same lung volume [10]. This method that, however, should be performed by body plethysmography to avoid artifacts due to the thoracic gas compression [11] is fatally flawed by the sequential emptying of the lung regions with uneven time constant and by different time and volume history of the lung parenchyma and airways in the preceding inspiration [12, 13]. In fact all these factors influence the corresponding expiratory flow rates that are going to be compared in the two maneuvers. To respect time and volume history with similar lung-emptying sequence and to limit (or avoid by using body plethysmography) thoracic gas compression, comparison between submaximal (i.e., with gentle expiratory effort) and resting tidal flow-volume curve has been suggested for assessing EFL. Obviously this technique demands high cooperation and uncommon ability from the patients and cannot be standardized. 

More than 15 years ago, to overcome all these problems, the Negative Expiratory Pressure (NEP) method has been introduced in the research and clinical practice [14]. A negative pressure of few cmH_2_O (usually 5 cmH_2_O) is applied at the mouth at the beginning of expiration to establish a pressure gradient between the alveoli and airway opening. During NEP that lasts for the whole expiration, there is an increase in expiratory flow in the absence of EFL, while the expiratory flow does not increase over the flow of the preceding control expiration, throughout the entire or part of the tidal expiration, in the presence of (total or partial) EFL (Figure 1). The NEP method that has been validated by using isovolume pressure-flow curves [15] does not require cooperation from the subjects and use of body plethysmography, can be performed at rest in any body position and during effort, and usually is devoid from interpretative problems. The only limit is the upper airway collapse possibly induced by the NEP application, as observed in snorers and OSAH patients, that can be partially controlled by reducing the negative pressure and repeating the measurements. The excessive spontaneous breath-to-breath changes in EELV can, however, lead to unclear results by using this technique.

This inconvenience is absent during the manual compression of abdominal wall (CAM) that, performed at rest or during exercise simultaneously with the start of tidal expiration, allows to increase expiratory flow rates over those of the preceding control expiration in the absence of EFL. In contrast, failure to increase expiratory flow rates during CAM indicates EFL [16]. The ability of the physician or technician, the cooperation of the patients, and the glottic reflex possibly elicited by this maneuver that cannot be standardized limit the utility of CAM for assessing EFL.

Recently the use of forced oscillation technique (FOT) during tidal breathing has been used to detect EFL breath-by-breath, both at rest and during exercise [17]. Briefly, when the oscillatory pressure applied at the mouth does not reach the alveoli during expiration because a flow limiting segment is present in the bronchial tree, the reactance signal, instead of reflecting the mechanical properties of the lung parenchyma and airways, is influenced only by those of the airways and becomes much more negative with a clear within-breath distinction between inspiration and expiration. This application of the FOT is very promising to identify EFL during tidal breathing, but the closure of intrathoracic airways eventually occurring at EELV must be considered as an important limiting factor of this technique, because the distortion of the reactance signal is similar.

## 4. EFL, Dynamic Hyperinflation, and Dyspnea

The development of EFL is functionally relevant because under the prevailing conditions (e.g., during exercise or at rest either in the supine or seated position) EFL is associated or promotes dynamic pulmonary hyperinflation (DH) by fixing, for a given expiratory tidal volume, the time required for the respiratory system to reach its relaxation volume (*V*_*r*_) [18]. Indeed, in the presence of EFL at rest, although DH can be avoided if the expiratory time is long enough, EELV is more often dynamically raised [19] and invariably increases with increasing ventilatory request (greater tidal volume and faster respiratory rate) [20]. When EFL develops during exercise, EELV starts to increase and inspiratory capacity to decrease, both signaling the occurrence of progressively greater DH [21].

DH promotes neuromechanical dissociation and implies a positive alveolar end-expiratory pressure (PEEPi) with a concomitant increase in inspiratory work, due to PEEPi acting as an elastic threshold load, impairment of the inspiratory muscles function, and adverse effects on hemodynamics [22]. These factors together with dynamic airway (downstream from the flow-limiting segment) compression during expiration may contribute to the dyspnea sensation [23, 24].

## 5. Clinical Aspects

In healthy subjects EFL occurs neither at rest nor during strenuous exercise [25], with the exception of highly fit old individuals in whom EELV tends to increase at high levels of exercise because of elevated values of minute ventilation they can reach before stopping [26]. Since maximal expiratory flow rates are reduced near EELV because of lung volume functional reduction due to age-related increase of closing capacity, EFL may develop under these circumstances [3]. Recently, however, for the same reasons EFL has been found by using the NEP technique also at rest in a large number of very old subjects, especially in small sized elderly women. Among these aged subjects chronic dyspnea was frequently reported in the absence of obvious cardiopulmonary diseases [27].

EFL may occur during tidal breathing at rest in COPD patients and has been found in more than 50% of the patients with moderate-to-severe-to-very-severe airway obstruction [6, 14, 19, 28]. Despite this general picture, changes in conventional indices of airway obstruction such as FEV_1_, PEF, and FEV_1_/FVC derived from maximal flow/volume curve are not useful to predict EFL, and special techniques must be adopted to accurately detect EFL in these patients [7]. In COPD EFL at rest has been found to correlate with chronic dyspnea better than routine spirometric parameters [7]. In fact, EFL more than airway obstruction *per se* entails a greater risk of dynamic pulmonary hyperinflation (DH), and DH has been recognized as an important cause of dyspnea either during exercise or at rest, due to its negative consequences on work of breathing, inspiratory muscle function, and, above all, neuromechanical coupling [21, 23]. 

**Figure 3:**
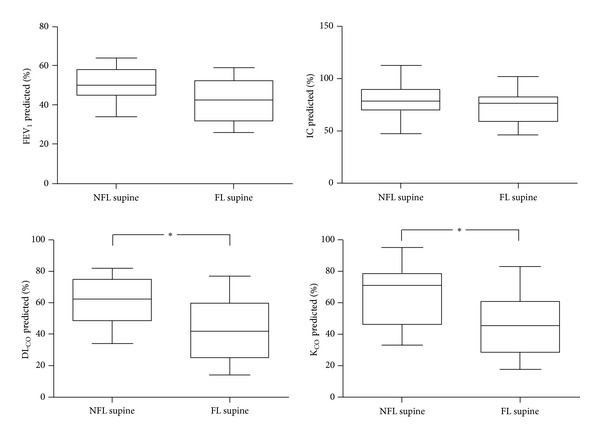
Comparison of FEV_1_, IC, DL_CO_, and K_CO_ in COPD patients who exhibit tidal expiratory flow limitation (EFL) in the supine position (FL; *n* = 14) versus those who do not (NFL; *n* = 13). Both DL_CO_ and K_CO_ are significantly lower in FL patients (*P < 0.05), suggesting that emphysematous patients are more prone to develop recumbent EFL.

It has been postulated that, in COPD for similar degrees of airflow obstruction, as measured by FEV_1_ reduction as percent predicted, EFL could be more easily observed, both during exercise and at rest, in patients with emphysematous phenotype in whom reduction of lung elastic recoil and loss of airway-lung parenchyma interdependence are thought to be the main determinants of airflow reduction. Under these conditions the peripheral small airways should be more compliant and prone to collapse during expiration favoring EFL that might partly explain the greater dyspnea reported by pink puffers. Recently, in a cohort of stable COPD patients with moderate-to-severe airflow obstruction, EFL assessed by the NEP technique was detected significantly more in those with lower values of DL_CO_ and K_CO_, but only when appraised in the supine position, suggesting an earlier appearance of EFL in emphysematous COPD patients (Figure 3). Interestingly, in these patients, chronic dyspnea, as measured by the modified MRC scale, was significantly greater (personal data). Further studies are needed to confirm this observation than links supine EFL and emphysema phenotype (pink puffer) in broader groups of COPD patients. 

During episodes of acute exacerbation and respiratory failure, COPD patients are prone to develop DH even in the absence of EFL because of increase in airway resistance with longer time constant in the respiratory system and rapid and shallow breathing with reduction of expiratory time [29]. Moreover, higher ventilatory requirements due to fever and/or anxiety, increased physiological dead space, and deterioration of gas exchange may contribute to DH. In the presence of EFL, however, all these factors cause a catastrophic increase in DH that cannot be longer sustained during spontaneous breathing without unbearable dyspnea and risk of acute fatigue of the respiratory muscles, leading to acute ventilatory failure (ARF) and adoption of mechanical ventilation [30]. With this regard, it should be stressed that almost all COPD patients mechanically ventilated for ARF exhibit EFL, since further increase in expiratory flow resistance is induced by endotracheal tube and expiratory circuit of the ventilator [31]. This is relevant when assisted mechanical ventilation is started because under these circumstances the inspiratory work could be very high yet, and the application of PEEP to counterbalance PEEPi can reduce the elastic threshold load without increasing EELV.

Conversely, apart from patients with severe chronic asthma who have uninterrupted, long-lasting, marked airway obstruction [7], EFL at rest is seldom observed in asthmatic patients, unless under severe and prolonged broncho-constriction [32].

In clinically stable patients with restrictive ventilatory disorders EFL is very uncommon during tidal breathing at rest [33]. 

In obese subjects and in patients with stable chronic heart failure EFL at rest is rarely present in seated position. However, recent studies showed that in massive obese subjects and patients with acute worsening of chronic congestive heart failure of EFL was frequently detected in the supine position [34, 35]. In all instances the development of EFL with recumbency prevents EELV to reach supine *V*_*r*_, leading to supine DH with concomitant PEEPi. Since this elastic threshold load imposed to shorter (and functionally weaker) inspiratory muscles has been related to dyspnea sensation, the occurrence of supine EFL may be associated with the onset of orthopnea either in massively obese subjects and patients with chronic heart failure [34, 35].

## 6. Conclusions

EFL is a very important mechanical constraint that frequently occurs in COPD patients, even with mild-to-moderate airflow obstruction, during exercise, fatally inducing the onset of DH and its progressive worsening, with the well-known negative mechanical, muscular, cardiovascular, and symptomatic consequences. Even worse in the natural history of COPD is the presence of EFL at rest, initially only in the supine position, contributing to orthopnea (and probably to more severe symptoms in early morning) in these patients and subsequently in the sitting-standing position limiting their daily physical activity and causing (very often) DH during resting tidal breathing with persistent volume-related mechanical stress in the lung parenchyma. Physicians who take care of COPD patients should be aware of this severe functional condition that, once established, rarely can be reversed with the present educational, pharmacological, and rehabilitative therapy and try to avoid it treating much earlier and more aggressively airflow obstruction and its determinants.
